# Development and Validation of an Electronic Health Record-Derived Prediction Model for Preventing COVID-19 Hospitalization and Death

**DOI:** 10.1007/s11121-025-01844-5

**Published:** 2025-10-20

**Authors:** R Yates Coley, Rachel Hays, Roy E. Pardee, Sharon Fuller, Kristine Rogers, Claire L. Allen, David E. Arterburn, Robert K. Frazier, Daniel J. Kent, Sophia Mun, Tolani Mwatha, Paul Thottingal, Emily O. Westbrook

**Affiliations:** 1 Kaiser Permanente Washington Health Research Institute, Seattle, WA USA; 2 Department of Biostatistics, University of Washington, Seattle, WA USA; 3 Department of Medicine, University of Washington, Seattle, WA USA; 4 Kaiser Permanente Washington, Seattle, WA USA; 5 School of Pharmacy, University of Washington, Seattle, WA USA

**Keywords:** Algorithm fairness, Clinical prediction model, Health equity, Learning health system, Nirmatrelvir/ritonavir (Paxlovid®), Prospective validation

## Abstract

Hospitalization and death following COVID-19 infection continue to pose a major public health concern and place strain on health system resources. Outpatient antiviral medication can reduce the risk of COVID-19 hospitalization and death for those at risk of poor outcomes, but identifying high-risk populations who may benefit most from treatment is challenging. The objective of this study was to develop and validate a prediction model for the composite outcome of hospitalization or death in the 14 days following COVID-19 infection. Our sample included 67,530 COVID-19 infections documented in outpatient care and occurring between April 1, 2020, and November 1, 2022, for 64,529 Kaiser Permanente Washington patients who did not receive outpatient antiviral treatment; 1378 (2.0%) of these infections resulted in hospitalization or death. Our prediction model, estimated using logistic regression with LASSO variable selection and ridge penalization, included 19 risk factors and showed high performance, including an area under the curve of 0.825 (95% confidence interval 0.813–0.836). Among the 10% of infections with the highest risk predictions, the true positive rate was 48% (46–51%) and the positive predictive value was 9.9% (9.2–10.6%). Supplemental analyses confirmed strong model performance across racial and ethnic subgroups and over time. We also present our process for selecting a risk threshold above which to recommend antiviral treatment and discuss considerations for prospective clinical implementation. This project demonstrates that machine learning tools can be used by health systems to deliver timely, targeted secondary prevention to reduce the risk of serious illness or death.

**Supplementary Information:**

The online version contains supplementary material available at https://doi.org/10.1007/s11121-025-01844-5.

## Introduction

The public health and health system burden of the COVID-19 pandemic has been substantial (Bartsch et al., 2020; French et al., 2021; Ledesma et al., 2024). At the height of the pandemic, in the United States, hospitalization rates were as high as 35.5 per 100,000 people (January 8, 2022) and death rates were as high as 25,974 weekly (January 9, 2021) (CDC, 2020). While more recent COVID-19 variants pose lower risk of serious complications than earlier ones and vaccines provides additional protection (Moore et al., 2025), infection with COVID-19 remains a leading cause of hospitalization and death and continues to pose a public health threat and strain on health systems in the US (Ahmad, 2023; WHO, 2024). In 2024, the Centers for Disease Control (CDC) estimated that 46,582 people died from COVID-19-related complications, and there were 72,000–120,000 COVID-19-associated hospitalizations from October 1 through December 7^th^, the first 10 weeks of the 2024–2025 respiratory virus season (CDC, 2025; CDC. Provisional COVID-19 Mortality Surveillance, 2025). COVID-19 has represented greater mortality and hospitalizations than seasonal influenza since its onset in 2020, with influenza deaths (1.7%) only surpassing COVID-19 deaths (1.4%) for the first time in 2025, during an exceptionally burdensome flu season (CDC. Weekly US Influenza Surveillance Report: Key Updates for Week 17, 2025).

Disparities persist in COVID-19 morbidity and mortality with American Indian or Alaska Native, Latino, Black, and Asian or Pacific Islanders experiencing higher rates of infection, hospitalization, and death from COVID-19 than non-Hispanic Whites in the US (Shortreed et al., 2023). Age-adjusted rate ratios (RR) of hospitalization range from 3.7 for American Indian or Alaska Native compared to White people to 1.03 for Asian or Pacific Islander compared to White people (Acosta et al., 2021; Dai et al., 2021; Ko et al., 2023; Mackey et al., 2021). Mortality rates follow this same trend, with American Indian or Alaska Native people having a death RR of 7.19, Latino people a RR of 3.85, Black people a RR of 2.58 and Asian or Pacific Islanders a RR of 1.64 compared to White people.

Antiviral medications administered in an outpatient setting can substantially reduce risk of COVID-19 hospitalization and death in high-risk populations (Cha-Silva et al., 2024). In particular, Paxlovid® (nirmatrelvir/ritonavir) reduced hospitalization and death by 89% in the clinical trial that led to its authorization by the FDA for emergency use in December 2021 (Hammond et al., 2022). Post-market studies using real-world observational data have shown 33–85% reductions in 30-day hospitalization and up to 77% reductions in all-cause mortality (Bajema et al., 2023; Cha-Silva et al., 2024; Shah, 2022). Subsequently, in May 2023, when the FDA approved the new drug application, Paxlovid® was approved for use for the treatment of mild-to-moderate COVID-19 in adults who are at high risk for progression to severe COVID-19 (FDA, 2024; Pfizer, 2025).

However, there are clinical and operational challenges to administering timely outpatient antiviral treatment to effectively prevent hospitalization or death. Treatment with Paxlovid® must be initiated within 5 days of symptom onset and the cost for a course of treatment is considerable, with the Average Wholesale Price (AWP) for Paxlovid® in the US being approximately $1500 for a 5-day course (GoodRx, 2025). Additionally, Paxlovid® has side effects—dysgeusia, diarrhea, and nausea being most common—( Hammond et al., 2024) and significant drug interactions that may require pharmacist management. This balance of benefits and harms poses a clinical dilemma for patients, providers, and health systems and underscores the need to quickly and accurately identify patients at higher risk of poor outcomes. However, identifying “high-risk” populations is challenging; the list of medical conditions and factors associated with increased risk for progression to severe COVID-19 is lengthy, evidence supporting various risk factors is mixed, and it is difficult to quantify the cumulative effect of multiple risk factors to accurately estimate an individual’s risk. These challenges are especially pronounced for patients with comorbidities who are 18–64 years old, whose overall risk of poor outcomes is lower than for older adults. Additionally, disparities in access to treatment persist with studies finding that non-Hispanic Black and Latino patients are significantly less likely to receive Paxlovid® compared to non-Hispanic White patients (Boehmer et al., 2022; Xiao et al., 2024). Many factors contribute to these inequities, including structural racism and its far-reaching effects on health, including limited access to healthcare and economic disadvantages (Acosta et al., 2021; Boehmer et al., 2022).

At the individual level, patients who believe that they are at lower risk of serious complications may decide that the cost, side effects, or potential for viral rebound from Paxlovid® do not warrant the potentially small benefit from treatment (The NNT, 2025; Smith-Jeffcoat et al., 2024). However, some patients may self-assess their risk-level as low even when they could benefit from Paxlovid®. All patients at high risk of progression to hospitalization or death should be counseled accurately on the potential preventive benefits of Paxlovid®. To inform clinical care decisions, providers need more specific guidance on which patients benefit most from Paxlovid®.

This study aimed to support clinical decision making about Paxlovid® prescribing at Kaiser Permanente Washington (KPWA) by developing and validating a prediction model to identify patients at higher risk of hospitalization or death following COVID-19 infection, with a focus on enabling providers to have data-informed conversations with patients about the potential benefits of Paxlovid® treatment. Before this study, KPWA prioritized Paxlovid® treatment for patients 65 years and older or patients with chronic lung disease or conditions that cause immunosuppression, either by diagnosis or treatment. This study was designed to address health system leaders’ interest in identifying additional patient populations who would benefit from treatment. In this paper, we describe the development and validation of a prediction model for COVID-19 hospitalization and death which is currently in use at KPWA. Model assessment prior to deployment included an examination of potential impacts on racial and ethnic disparities in COVID-19 treatment and an analysis of the model’s robustness to time-varying trends in COVID-19 infections and outcomes. This paper also describes the selection of a clinical threshold for classifying infections among 18–64-year-olds as high-risk for hospitalization and death, thereby expanding Paxlovid® treatment prioritization beyond the original high-risk groups.

## Methods

### Setting

This study was conducted at KPWA, an integrated health system providing medical care and insurance coverage to more than 400,000 patients in Washington and Idaho. The KPWHRI IRB determined this quality improvement initiative was not research (IRB#1,972,759).

### Population

The study sample included COVID-19 infections documented in clinical records or health insurance claims data and occurring between April 1, 2020, and November 1, 2022, for patients aged 18 years and older paneled to a KPWA primary care provider. COVID-19 infections were identified by the presence of a COVID-19 diagnosis code, positive PCR test, or patient self-report of a positive at-home test via the electronic patient portal. (Throughout the manuscript “infection” refers to infection with COVID-19.) The date of the first documented evidence of infection was defined as the “index date.” COVID-19 infections that were first documented on the day of hospital admission or during an inpatient hospitalization were excluded from the sample because the outcome of interest (hospitalization) had already occurred. Analyses also excluded infections that received prescription outpatient antiviral treatment, including Paxlovid®, Lagevrio® and Veklury®, as these treatments alter hospitalization risk. Patients could have multiple COVID-19 infections included in the study sample so long as the subsequent infection occurred at least 90 days after the immediately prior documentation of COVID-19 diagnosis or positive test.

### Outcome

The outcome for prediction was a composite of all-cause inpatient hospitalization or all-cause mortality within 14 days of the infection index date. Outcome events were not limited to COVID-19-related hospitalizations or death as preliminary analyses indicated inconsistent relevant documentation. Dates of hospitalization and death were obtained from insurance claims data.

### Predictors

Potential risk factors for hospitalization or death following COVID-19 infection were assessed as of the index date. These predictors included demographics (age, sex, race, ethnicity); insurance information; comorbidity indicators, including components of the Gagne comorbidity index (Gagne et al., 2011) and comorbidities hypothesized to be related to COVID-19 outcomes; Johns Hopkins Adjusted Clinical Group (ACG) estimated 12-month hospitalization risk; healthcare utilization in the prior year, including outpatient, inpatient, emergency department and urgent care encounters; COVID-19 related risk factors, such as number and timing of prior vaccinations, number of prior COVID-19 infections, and type of infection documentation (PCR test, diagnosis, or at-home test); body-mass index; patient-reported tobacco use; and current pregnancy status. The resulting candidate predictor set included over 100 variables. Predictor data from KPWA’s research data warehouse (Ross et al., 2014) (containing clinical records and health insurance claims on all KPWA patients) and from the electronic health record (EHR) (containing clinical records) were used in analyses, as described below.

Since eventual implementation of the prediction model would occur within the EHR, clinical records data would be the only available source for calculating risk scores in real-time. However, defining predictor variables in the EHR for over 100 potential risk factors for our initial model development was time prohibitive. Thus, for Phase 1 of model development, predictor data were collected from a research data warehouse—identifying existing variables in that environment is much faster—and variable selection was conducted to identify a subset for predictors for the final prediction model estimation (Phase 2).

### Statistical Analysis

The goal of analysis was to use predictor data available in the EHR to accurately predict risk of COVID-19 hospitalization or death following infection and to use these predictions to distinguish between infections at higher and lower risk. To accomplish this, ten-fold cross-validation was used for variable selection, model estimation, and validation to maximize the sample size available for both tasks (Coley et al., 2023; Steyerberg et al., 2001). Following this approach, infections were randomly separated into 10 independent subgroups or *folds*. Each fold was iteratively used as the held-out *testing* set for validating a prediction model estimated in the *training* set comprised of the remaining 9 folds.

### Model Development Phase 1 – Variable Selection

The objective of Phase 1 was to perform variable selection to identify the most important predictors or COVID-19 hospitalization and death from over 100 candidate predictors using logistic regression with LASSO (least absolute shrinkage and selection operator) (Tibshirani, 1996). LASSO is a method that prevents overfitting by selecting the predictors most strongly associated with the outcome (conditional on other selected predictors) and shrinking or penalizing coefficient estimates towards no association. Within each training set, LASSO was used to identify the penalization parameter that optimized performance in the testing set, as measured by the area under the receiver operation characteristic curve (AUC). The optimal penalization parameter was then applied to the entire dataset to select predictors to be included in the final prediction model.

In response to concerns that minoritized racial and ethnic populations were less likely to access needed treatment, we explored the impact of estimating our logistic regression model without any LASSO penalty on a composite race and ethnicity variable indicating whether a patient had a documented race or ethnicity other than White, non-Hispanic in their records; that is, we required this variable to be included in the model and did not shrink the coefficient estimate towards zero. We used a composite variable that grouped together all patients of with a race or ethnicity other than White non-Hispanic into a single binary indicator of “patients of color” as we were concerned that sample size in some minority groups would be insufficient to accurately inform prediction. We ultimately selected this modeling approach (instead of a model without race and ethnicity information), as it maintained high overall performance and increased risk predictions for racial and ethnic groups that experience barriers to outpatient treatment.

### Model Development Phase 2 – Application to the EHR Environment

After variable selection occurred in Phase 1, we then defined a subset of implementable predictors (coinciding with the predictors selected by the LASSO) for construction in the EHR environment in Phase 2. The final Phase 2 model was fit with EHR-derived predictors using ridge regression for coefficient shrinkage—in contrast to LASSO, ridge only penalizes coefficient estimates but does not perform additional variable selection. This approach was chosen to maintain the LASSO-selected variables’ inclusion in the prediction model while using ridge penalization to avoid overfitting. The optimal degree of shrinkage was selected by cross-validation.

### Evaluation of Model Performance

Performance of the prediction model trained with EHR-derived predictors was evaluated in the respective testing sets for each iteration of cross-validation and averaged across iterations to obtain estimates of performance. Performance measures assessed include AUC and classification accuracy (true positive rate [TPR], false positive rate [FPR], positive predictive value [PPV], and negative predictive value [NPV]) at the 95^th^, 90^th^, 75^th^, 50^th^, and 25^th^ percentiles of the risk score distribution. Bootstrap resampling was used to calculate quantile-based 95% confidence intervals (CIs) around performance estimates. Each bootstrap replication sampled infections with replacement within each cross-validation fold, evaluated performance measures within each fold’s bootstrap sample, and averaged performance estimates across folds. Predictive performance was summarized in the overall sample and within subgroups defined by age (18–64 years old vs. ≥ 65 years old) and by race and ethnicity.

### Evaluation of Prospective Validity

Since this project aimed to develop and validate a prediction model that would ultimately be used prospectively to guide Paxlovid® prescribing, a sensitivity analysis was also conducted to ensure the robustness of model performance over time. Specifically, we examined whether predictions estimated in a sample of COVID-19 infections from earlier time frames using the methods described above would accurately predict risk of hospitalization and death in future infections. To evaluate this prospective validity, we iteratively estimated a prediction model with earlier data to validate in the following 2 months, starting with a model estimated in March-September 2020 infections validated in October–November 2020 infections, followed by model training in March-November 2020 infections and validating in December 2020-January 2021, etc.

### Assessment of Risk Thresholds for Clinical Implementation

To respond to health system leaders’ interest in expanding identification of patients who may benefit from antiviral treatment, we completed additional analyses in the subset of the sample not already recommended for treatment (i.e., age 18–64 years old and without chronic lung disease or conditions that cause immunosuppression) to assess potential thresholds for classifying risk following COVID-19 infection. Analyses were additionally restricted to the more recent infections in the sample, from April 1- November 1, 2022, to better reflect the clinical population in 2023 (when this model was first implemented). We evaluated TPR and PPV at a range of possible thresholds (5–50%, in increments of 5%) as well as the event rate within separate risk strata (defined at every 5th percentile) to quantify the potential benefit of expanding high-risk classification.

Additional details about model predictors, data collection, and analytic methods, including the CHecklist for critical Appraisal and data extraction for systematic Reviews of prediction Modelling Studies (CHARMS) (Moons et al., 2014), are included in the supplementary material. Data collection and statistical analysis were conducted from January-August 2023.

## Results

### Sample

We identified 67,530 eligible COVID-19 infections documented for 64,529 patients between April 1, 2020, and September 30, 2022. Sample characteristics are described in Table 1. One-third of infections were documented by diagnosis code and 61% were documented by PCR test, while only 14% of infections were indicated by patient self-report of an at-home test. (Diagnoses could be documented by multiple sources on the same day.) Twenty-one percent of documented infections were in adults 65 years and older. (By comparison, adults 65 years and older comprised 28% of the adult KPWA patient population.) Over 60% of infections were documented in 2022, corresponding to the dominance of the Omicron variant, which showed a higher rate of transmissibility than prior variants.

**Table 1:** Characteristics of sample for prediction study

	All infections	Infections without event	Infections with event	Event rate^1^
Characteristic	*N* = 67,530	*N* = 66,152	*N* = 1,378	
Age, years median (IQR)	48 (35–62)	48 (35–62)	61 (46–74)	
18–29	10,264 (15.2%)	10,193 (15.4%)	71 (5.2%)	6.9
30–39	13,175 (19.5%)	13,014 (19.7%)	161 (11.7%)	12.2
40–49	12,168 (18.0%)	11,995 (18.1%)	173 (12.6%)	14.2
50–59	12,025 (17.8%)	11,784 (17.8%)	241 (17.5%)	20.0
60–69	10,852 (16.1%)	10,557 (16.0%)	295 (21.4%)	27.2
70–79	6545 (9.7%)	6330 (9.6%)	215 (15.6%)	32.8
80 and older	2501 (3.7%)	2279 (3.4%)	222 (16.1%)	88.8
Sex				
Male	28,643 (42.4%)	27,973 (42.3%)	670 (48.6%)	23.4
Female	38,887 (57.6%)	38,179 (57.7%)	708 (51.4%)	18.2
Race^2^				
American Indian/Alaskan Native	1045 (1.5%)	1011 (1.5%)	34 (2.5%)	32.5
Asian	7437 (11.0%)	7304 (11.0%)	133 (9.7%)	17.9
Black	4474 (6.6%)	4377 (6.6%)	97 (7.0%)	21.7
Native Hawaiian/Pacific Islander	1314 (1.9%)	1284 (1.9%)	30 (2.2%)	22.8
White non-Hispanic	42,742 (63.3%)	41,867 (63.3%)	875 (63.5%)	20.5
Hispanic ethnicity	5355 (7.9%)	5247 (7.9%)	108 (7.8%)	20.2
No recorded race or ethnicity	4905 (7.3%)	4814 (7.3%)	91 (6.6%)	18.6
Medicare insurance	14,388 (21.3%)	13,786 (20.8%)	602 (43.7%)	41.8
Length of prior KPWA insurance enrollment				
< 1 year	14,806 (21.9%)	14,516 (21.9%)	290 (21.0%)	19.6
1–3 years	18,692 (27.7%)	18,386 (27.8%)	306 (22.2%)	16.4
3 years or more	34,032 (50.4%)	33,250 (50.3%)	782 (56.7%)	23.0
Calendar year				
2020	9237 (13.7%)	8843 (13.4%)	394 (28.6%)	42.7
2021	16,204 (24.0%)	15,588 (23.6%)	616 (44.7%)	38.0
2022	42,089 (62.3%)	41,721 (63.1%)	368 (26.7%)	8.7
First documentation of infection^3^				
COVID-19 diagnosis	22,281 (33.0%)	21,697 (32.8%)	584 (42.4%)	26.2
Positive PCR test	41,054 (60.8%)	40,094 (60.6%)	960 (69.7%)	23.4
Positive at-home test	9672 (14.3%)	9657 (14.6%)	15 (1.1%)	1.6
Number of COVID-19 vaccines				
0	19,868 (29.4%)	19,002 (28.7%)	866 (62.8%)	43.6
1	3497 (5.2%)	3414 (5.2%)	83 (6.0%)	23.7
2	17,211 (25.5%)	16,994 (25.7%)	217 (15.7%)	12.6
3 or more	26,954 (39.9%)	26,742 (40.4%)	212 (15.4%)	7.9

IQR: Interquartile Range

^1^Rate per 1,000 infections of hospitalization or death within subset of sample with that characteristic (e.g., infections in female patients) reported for categorical variables

^2^Race and ethnicity data are collected by patient self-report and follow United States Office of Management of Budget designations. Categories are not mutually exclusive

^3^Categories are not mutually exclusive as infections could be documented by more than one route on the index date

### Hospitalization and Mortality Outcomes

We observed 1,310 hospitalizations and 110 deaths within 14 days of infection for an event rate of 19 hospitalizations and 1.6 deaths per 1,000 infections. The composite outcome of hospitalization or death within 14 days was observed for 1,378 (2.0%) of infections (Table 1). Forty-one percent of events were observed in older adults, and older adults had a 2.7 times higher risk of 14-day hospitalization or death than adults 18–64 years old (4.15% vs. 1.5% event rate). While most infections were documented in 2022, only 21% of hospitalizations and deaths occurred in 2022 infections. Event rates for infections in 2020 and 2021 were 4–5 times higher than those that occurred in 2022.

### Prediction Modeling Results

Our Phase 1 modeling approach selected 19 predictors of COVID-19 hospitalization and death including age, vaccination history, and comorbidities. Table 2 presents the log-odds coefficient estimates from the Phase 2 logistic regression model with ridge penalization estimated using EHR-derived predictors.

**Table 2:** Selected risk factors for prediction model, coefficient estimates for penalized logistic regression model, and summary of risk factors in sample

	Coefficient	All infections	Infections without event	Infections with event	Event rate^1^
	Estimate	*N* = 67,530	*N* = 66,152	*N* = 1,378	
Intercept	−4.948	–	–	–	–
Age, years^2^	0.011	48.8 (17.3)	48.5 (17.2)	59.9 (18.3)	–
Age ≥ 80	0.471	2501 (3.7%)	2279 (3.4%)	222 (16.1%)	88.8
Documented race or ethnicity other than White non-Hispanic^3^	0.110	19,883 (29.4%)	19,471 (29.4%)	412 (29.9%)	20.7
ACG 12-month hospitalization risk^2^	0.272	0.45 (0.69)	0.44 (0.66)	1.18 (1.4)	–
ACG missing^4^		3383 (5.0%)	3319 (5.0%)	64 (4.6%)	18.9
> 10 outpatient visits in prior year	0.201	6359 (9.4%)	6128 (9.3%)	231 (16.8%)	36.3
BMI ≥ 30 kg/m^2^	0.224	29,605 (43.8%)	28,801 (43.5%)	804 (58.3%)	27.2
Diagnoses					
Autoimmune diagnosis	0.199	1872 (2.8%)	1798 (2.7%)	74 (5.4%)	39.5
Chronic kidney disease	0.403	2707 (4.0%)	2472 (3.7%)	235 (17.1%)	86.8
Chronic lung disease	0.273	1418 (2.1%)	1311 (2.0%)	107 (7.8%)	75.5
Congestive heart failure	0.252	1184 (1.8%)	1060 (1.6%)	124 (9.0%)	104.7
Dementia	1.170	266 (0.4%)	210 (0.3%)	56 (4.1%)	210.5
Diabetes	0.369	7530 (11.2%)	7154 (10.8%)	376 (27.3%)	49.9
Hepatitis	0.251	603 (0.9%)	577 (0.9%)	26 (1.9%)	43.1
Immunosuppressed	0.171	1334 (2.0%)	1274 (1.9%)	60 (4.4%)	45.0
Peripheral vascular disease	0.404	764 (1.1%)	685 (1.0%)	79 (5.7%)	103.4
COVID-19 related					
COVID-19 infection documented by diagnosis code (vs. test)	0.053	22,281 (33.0%)	21,697 (32.8%)	584 (42.4%)	26.2
No prior vaccination	0.624	19,868 (29.4%)	19,002 (28.7%)	866 (62.8%)	43.6
Prior vaccinations, count^2^	−0.219	1.9 (1.4)	1.9 (1.4)	0.9 (1.3)	–
Only vaccination in past 2 weeks	1.196	493 (0.7%)	461 (0.7%)	32 (2.3%)	64.9

^1^Rate per 1,000 infections of hospitalization or death within subset of sample with that characteristic

^2^For continuous or count variables, the mean(standard deviation) is reported instead of counts and percentages. No event rate is presented

^3^Composite binary variable designating “patients of color.” Variable equals 1 for infections in patient with some self-reported race or ethnicity other than White non-Hispanic. If no race or ethnicity is reported, this variable equals 0

^4^For infections missing ACG, mean imputation was used for prediction model estimation, validation, and implementation

Our prediction model showed strong performance in the overall sample as well as in subgroups defined by age (Table 3). Cross-validated AUC was 0.825 (95% CI: 0.813–0.836) in the entire sample and 0.802 (0.787–0.818) in a subset of infections in 18–64-year-olds. When flagging the highest 10% of infections as high-risk for hospitalization or death, the model had a TPR of 48% (95% CI: 46–51%) in the entire sample and 47% (43–50%) among 18–64-year-olds. The PPV at this threshold was 9.9% (9.3–10.7%) in the entire sample and 7.1% (6.5–7.6%) among 18–64-year-olds, indicating that older adults in this stratum had greater average risk. Performance of the Phase 1 and 2 models was similar (Table S1).

**Table 3:** Performance of prediction model for risk of hospitalization or death in 14 days following COVID-19 infection

	All ages	Age 18–64 year olds
AUC	0.825 (0.813, 0.836)	0.802 (0.787, 0.818)
TPR^1^		
≥ 90^th^	0.484 (0.459, 0.511)	0.470 (0.434, 0.501)
≥ 75^th^	0.756 (0.732, 0.778)	0.724 (0.693, 0.754)
≥ 50^th^	0.900 (0.884, 0.914)	0.885 (0.863, 0.906)
FPR		
≥ 90^th^	0.092 (0.090, 0.094)	0.094 (0.092, 0.097)
≥ 75^th^	0.239 (0.236, 0.243)	0.243 (0.239, 0.246)
≥ 50^th^	0.491 (0.488, 0.495)	0.494 (0.490, 0.498)
PPV		
≥ 90^th^	0.099 (0.092, 0.106)	0.071 (0.065, 0.076)
≥ 75^th^	0.062 (0.058, 0.065)	0.044 (0.042, 0.045)
≥ 50^th^	0.037 (0.035, 0.039)	0.027 (0.026, 0.027)

(NPV is not included in Table 3, as estimates were over 0.98 for all thresholds and all subgroups.)

^1^Thresholds for classification accuracy measures were defined based on the distribution of risk scores among all infections (left hand column) or infections among 18–64 year olds (right hand column). For example, ≥ 90^th^ percentile refers to the risk prediction threshold that would classify the 10% of infections with the greatest risk as high-risk for hospitalization and death and, subsequently, classify the 90% of infections with a predicted risk below that threshold not high-risk

**Fig. 1:**
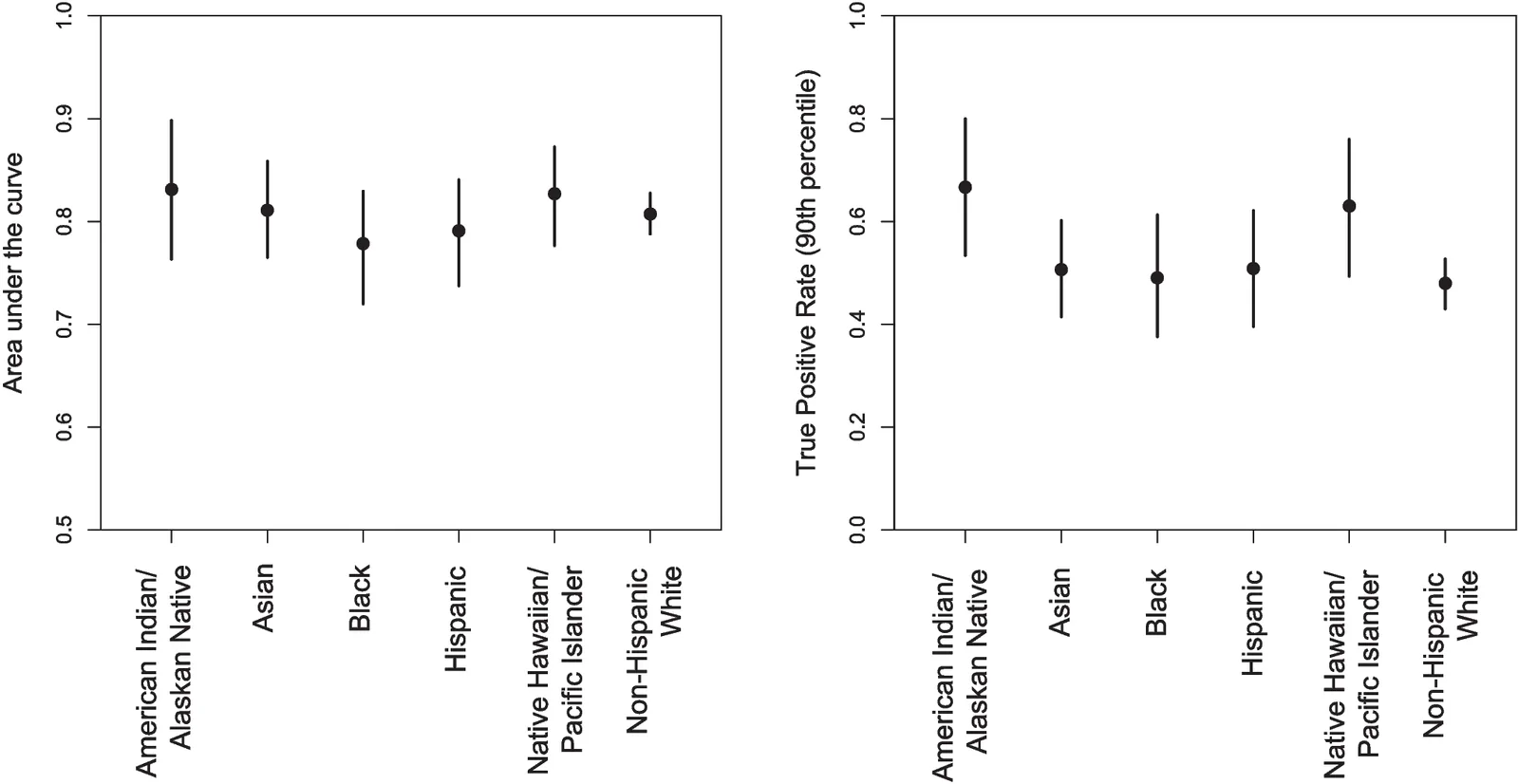
Prediction model performance for infections in 18–64 year olds within racial and ethnic subgroups as measured by AUC (left) and TPR when the 90^th^ percentile of the risk score distribution is used to classify high-risk visits (right)

Model performance in 18–64-year-olds was similar across racial and ethnic groups, with AUC estimates ranging from 0.78 to 0.83 (Fig. 1). We also observed adequately strong classification accuracy in all racial and ethnic groups in this age range (Tables S2–S4). For example, the TPR at the 90th percentile of risk predictions (i.e., among the 10% of infections at highest risk), is similar among Asian, Black, Hispanic, and non-Hispanic White patients with 48–51% of infections in these groups resulting in hospitalization or death. We observed higher TPRs at this threshold for American Indian/Alaskan Native and Native Hawaiian/Pacific Islander patients, which is expected for subgroups with higher event rates (as seen in Table 1).

### Evaluation of Prospective Validity

**Fig. 2:**
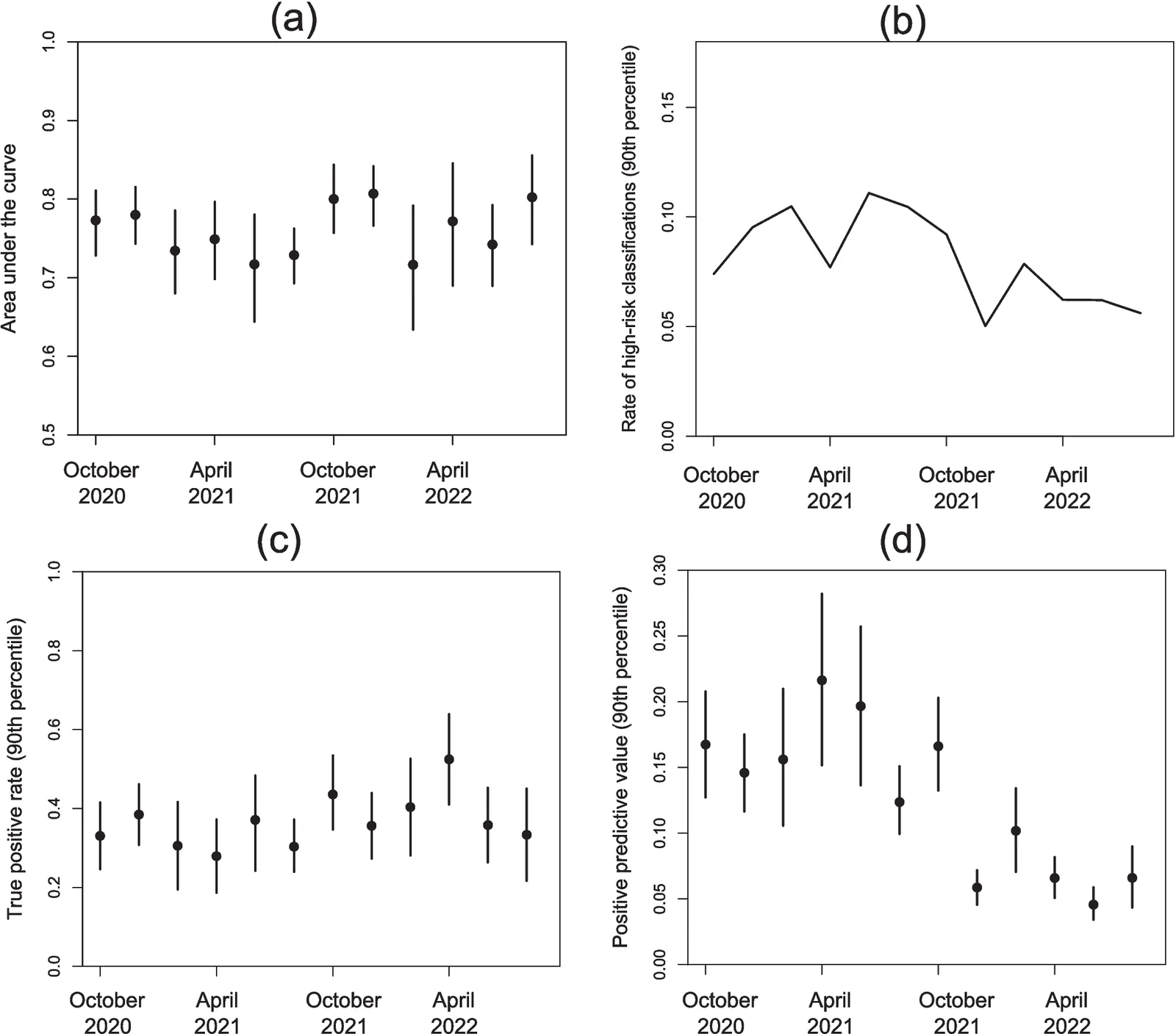
Prospective validation of modeling approach including (**a**) AUC, (**b**) proportion of infections with a predicted risk above the set threshold, (**c**) true positive rate, and (**d**) positive predictive value. In (b-d) the 90^th^ percentile threshold is defined by the risk score distribution in the retrospective training data

Our prospective validation analysis simulating future two-month performance of a prediction model estimated on retrospective data showed that AUC estimates were moderately robust to time trends in COVID-19 infections and outcomes, indicating that infections were similarly well-ordered with respect to risk over time (Fig. 2a). Classification accuracy measures, however, varied more meaningfully over time. Figure 2b–d demonstrates this variability at the 90th percentile of the risk distribution, that is, when the distribution of risk predictions in the retrospective sample was used to define a risk threshold above which 10% of infections would be classified as high-risk. We see that the proportion of infections flagged as high-risk varies considerably over time, remaining well-below the 10% target in the final year (Fig. 2b). TPR at this threshold ranges widely, from estimates below 30% to those above 50%, over the study period (Fig. 2c). There are also substantial time trends in PPV (Fig. 2c). From October 2020 to mid-2021, 15–22% of infections with predicted risk above the 90th percentile threshold resulted in hospitalization or death before PPV began to lower. In the last 6 months of the sample, PPV remained below 7%.

### Assessment of Risk Thresholds for Clinical Implementation

**Fig. 3:**
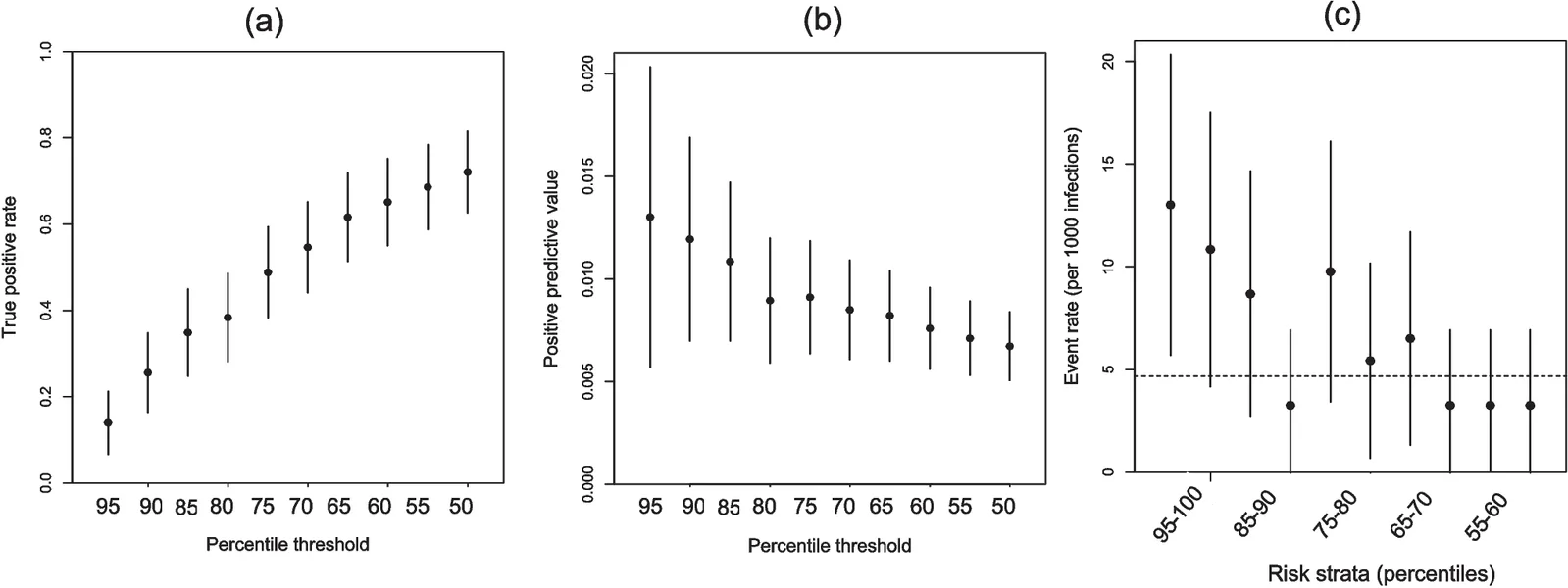
Assessment of potential percentile thresholds for classifying high-risk visits among infections in adults 18-64 years old and without immunocompromising conditions from April 1 – November 1, 2022. Panels (**a**) and (**b**) depict the TPR and PPV, respectively, at potential thresholds. Panel (**c**) shows the event rate at a range of risk strata; in this plot, the horizontal dashed line indicates the event rate in this population (4.8 per 1000 infections)

Our sample included 18,717 infections from April 1- November 1, 2022, among 18–64-year-olds without chronic lung or immunocompromising conditions (Table S5–S6). Only 89 events (including 1 death) were observed in this subsample, constituting an event rate of 4.8 hospitalizations or deaths per 1,000 infections. Figure 3 shows our assessment of ten possible risk thresholds above which infections would be labeled high-risk, ranging from 5% of infections with the highest risk (i.e., the 95^th^ percentile) to the top 50%. TPR increases from 15% at the 95^th^ percentile to 27% at the 90th percentile; each further addition of 5% of visits into the high-risk category yields less than 10% gain to the TPR (Fig. 3a). PPV is above 1% for thresholds defined at the 95^th^, 90^th^, and 85^th^ percentile of the distribution (Fig. 3b). Event rates within risk strata defined at every 5^th^ percentile of the risk distribution generally show the expected ordering of risk—higher risk strata have higher event rates—with some variability (Fig. 3c).

## Discussion

The COVID-19 pandemic continues to negatively impact individuals, population health, and health systems through the large number of people who progress to severe disease and death. Ambulatory treatments, such as Paxlovid®, provide the potential for secondary prevention of poor outcomes from COVID-19, but treating all infections with Paxlovid® would be logistically challenging and prohibitively expensive.

Having the ability to accurately predict risk of poor outcomes was identified early in the COVID-19 pandemic as a key strategy to inform rapid triage and guide decisions regarding treatment (Nicola et al., 2020). Several tools to predict hospitalization or death from COVID-19 have been developed since the onset of COVID-19. A 2022 systematic review of clinical prediction models for mortality in patients with COVID-19 demonstrated most of the tools demonstrate high predictive accuracy; however, the prognostic value of the existing models varied widely across populations and settings, and they require regular validation and updating (de Jong et al., 2022). This underscores the challenge of implementing a COVID-19 risk prediction model into routine clinical care.

To our knowledge, the prediction model described here is one of few that has been developed and validated in a more recent, vaccinated population and implemented at scale in a health system while accounting for temporal variability. Our model also focuses on preventing hospitalization or death in an ambulatory population, while many other risk prediction tools focus specifically on prediction of death among hospitalized patients (Hajifathalian et al., 2020; Hippisley-Cox et al., 2023; de Jong et al., 2022; Xu et al., 2022). The risk prediction model we’ve developed can be adapted and scaled to other health systems or agencies with access to clinical records data, as the logistic regression form enables easy updating to new settings and populations (Steyerberg, 2019; Saelmans et al., 2025)  Real-time risk predictions available in the EHR can be implemented into clinical workflow to guide patient selection for outpatient treatment to help prevent hospitalization and death and inform shared decision-making conversations between patients and providers. We envision that this information will encourage high-risk patients to receive treatment while dissuading those at lower risk to undergo treatment, avoiding unnecessary costs and side effects. Our project was additionally unique in that we took a collaborative approach with clinicians and health system leaders in developing and validating the risk prediction model.

This tool and approach also provide an opportunity to address the racial and ethnic disparities in infection, treatment, hospitalization and death that have been observed throughout the COVID-19 pandemic. It is important for treatment guidelines to take this context into account and for health systems to proactively design interventions to help address disparities. Our study used two approaches to ensure that clinical use of the risk prediction model would not exacerbate existing disparities in Paxlovid® treatment. First, we included race and ethnicity information in our prediction model to reflect increased risk of hospitalization and death for American Indian or Alaska Native, Asian or Pacific Islander, Black, and Hispanic patients. As a result, more non-White patients are flagged as high-risk and, subsequently, may be more likely to be offered and receive treatment. Second, we assessed prediction model performance across racial and ethnic subgroups to confirm comparable classification accuracy. These strategies established that implementation of the resulting model would correctly identify patients from minoritized racial and ethnic groups at higher risk of hospitalization and death and increase their access to risk-reducing treatment.

Clinical management of a rapidly evolving pandemic virus like COVID-19 presents distinct challenges for health systems. Factors such as disease understanding and predictability, diagnostics and treatment, severity and clinical burden and long-term health impacts are well understood for common, seasonal respiratory viruses such as influenza. This allows health systems to plan and implement interventions accordingly. However, COVID-19 continues to challenge our traditional public health and health system management strategies due to its rapidly evolving variants, the lack of predictability of surges, and the potential for unpredictable long-term sequelae from the disease.

In this setting where there are frequent shifts in dominant variants, transmission rates, infection severity, and individual susceptibility, a key concern is whether a prediction model trained with historical data will accurately identify high-risk visits in the future. We emulate this scenario in our analysis by iteratively training the model on earlier data and validating on future data to assess how robust prediction model performance may be if used prospectively. While we did observe some variability in risk discrimination, AUC remained acceptably high, indicating that similar risk factors are predictive of poor outcomes across variants. Classification accuracy at risk thresholds defined in the retrospective sample showed more clinically meaningful variability as strain severity and population risk changed over time. For example, the proportion of infections with predicted risk above a set threshold declined with each new wave of vaccination as the infected sample had protection conferred from more prior vaccinations. This variability must be managed after prediction model deployment to ensure that the proportion of infections classified as high-risk aligns with health system resources and that the right population is targeted for treatment given emerging understanding of the severity of a new strain. At KPWA, strategies that we use to navigate this variability include updating thresholds for high-risk classification based on recent risk score distributions, monitoring the proportion of infections flagged as high-risk, and recalibrating model predictions to provide accurate absolute estimates of patient risk.

This model was developed with the goal of expanding identification of adults at high-risk of poor outcomes following COVID-19 infection. To that end, we investigated several possible risk prediction thresholds that could be used to flag high-risk infections to recommend for consideration of antiviral treatment. Our analyses indicated that classifying the top 10% of infections (i.e., risk predictions greater than the 90th percentile) or top 15% of infections were promising strategies given high TPR relative to the proportion of infections flagged (27% and 35%, respectively) and PPVs greater than 1%. The marginal benefit of identifying a greater proportion of infections as high-risk declined after the 85th percentile. Ultimately, selecting a threshold for clinical implementation of the prediction model required considering the expected benefit of Paxlovid® in each risk strata as well as health system resources to offer timely, comprehensive clinical attention to all COVID-19 infections (including those among older adults and patients with immunocompromising conditions and chronic lung disease). KPWA leaders decided to categorize the top 10% of infections in this subsample as high-risk; this model is currently in use at KPWA.

This study has some limitations. First, our prediction model was trained and validated on the outcome of 14-day all-cause hospitalization and death, as it was not possible to distinguish COVID-19-related outcomes from hospitalizations and deaths due to other causes. Second, the study sample does not represent all infections among KPWA patients but rather includes only infections documented in outpatient settings via diagnosis or test. Thus, the sample excludes COVID-19 infections identified upon inpatient admission, infections identified with PCR tests or at-home tests performed outside of KPWA and not reported to a provider, and infections for which a patient did not receive a test. Testing patterns varied throughout the pandemic including shifting testing requirements for work, travel, or other activities and emerging availability of at-home tests. The prediction model presented here should be interpreted in light of these limitations. Coefficient estimates should not be used to draw generalized inferences about the relationship between model predictors and risk of hospitalization or death from COVID-19 in the broader population of all infections. Instead, the model should be interpreted within the context for which it was developed—estimating risk of any short-term hospitalization or death among KPWA patients accessing outpatient care for COVID-19 diagnosis and potential treatment.

## Conclusion

We successfully developed a novel prediction model for estimating the risk of hospitalization and death in the 14 days following COVID-19 infection. Our model was estimated with machine learning methods applied to EHR data from a large health system in Washington state. Our model showed high performance in the overall sample, within subgroups defined by race and ethnicity, and over time. This prediction model and its implementation at KPWA demonstrate how health systems can leverage advanced analytic methods and rich clinical data to inform individualized, evidence-based, equity-promoting prevention strategies and improve health outcomes.

## Supplementary Information

Below is the link to the electronic supplementary material.

Supplementary file1 (DOCX 104 KB)

## Data Availability

The dataset used in this study is not publicly available as it contains detailed information from patient clinical records data.
