# Supplementary material for: Development and Validation of an Electronic Health Record-Derived Prediction Model for Preventing COVID-19 Hospitalization and Death
Source: Prev Sci. 2025 Oct 20;27(7):1023–35. doi: 10.1007/s11121-025-01844-5 (PMC13550066; doi:10.1007/s11121-025-01844-5)
Supplement: Supplementary file 1 [file 11121_2025_1844_MOESM1_ESM.docx]

**Supplementary Material**

**CHARMS Checklist** can be found on final pages of the supplement

**Additional details of sample definition and data collection:**

**Sample Definition:**

*Sample:* All incident COVID infections since April 1, 2020 and before November 1, 2022 for IGP (Integrated Group Practice) KPWA members aged 18 or older (at index date).

*Unit of analysis:* Infection instance. People can have multiple infections.

*How to identify incident Covid infections?*

- Covid dx code (U07.1), OR
- Covid positive test (include PCR and at-home antigen tests), AND
- No covid dx code or positive test in the 90 days prior to index date

*Exclusion criteria:*

- Hospitalized on index date (including admissions prior to and on index date)
- Received outpatient antiviral treatment:
  - Molnupiravir
  - Remdesivir
  - Bebtelovimab
  - bamlanivimab plus etesevimab
  - casirivimab plus imdevimab
  - sotrovimab
  - nirmatrelvir/ritonavir

**Predictors considered for inclusion in prediction model:**

| **Variable** | **Notes** |
| --- | --- |
| Age | Age (in years, with 2 decimal places) at index date |
| Sex | Sex (administrative or “at birth”) |
| Race | All patient self-reported races |
| Hispanic | Patient-reported ethnicity |
| Insurance type | Medicare, Individual, Commercial, Medicaid |
| Length of enrollment | How many days continuous insurance enrollment (allow gaps up to 32 days) prior to index date |
| BMI | Indicator of BMI >= 30, most recent in prior 5 years |
| Tobacco use (2 vars) | Current and ever smoked status |
| Prior utilization (3 vars) | Count of number outpatient, emergency department/urgent care, and inpatient encounters in the year prior to index date. |
| COVID-19 vaccination status | Count of vaccinations prior to index date. |
| Days since COVID-19 vaccination | # of days between last pre-index vaccination and index date |
| Gagne individual comorbidity flags/indicators (many variables) | Full list of Gagne conditions:   1. Metastatic cancer 2. Congestive heart failure 3. Dementia 4. Renal failure 5. Weight loss 6. Hemiplegia 7. Alcohol abuse 8. Any tumor 9. Cardiac arrhythmias 10. Chronic pulmonary disease 11. Coagulopathy 12. Complicated diabetes 13. Deficiency anemias 14. Fluid and electrolyte disorders 15. Liver disease 16. Peripheral vascular disorder 17. Psychosis 18. Pulmonary circulation disorders 19. HIV/AIDS 20. Hypertension |
| ACG score | ACG Score that the provider would know in clarity at the index date. |
| Asthma |  |
| Bronchiectasisor COPD |  |
| Cerebrovascular disease |  |
| Chronic kidney disease |  |
| Cystic fibrosis |  |
| Interstitial lung disease |  |
| Pregnancy on index date |  |
| Primary immunodeficiencies |  |
| Solid organ or blood stem cell transplantation |  |
| Tuberculosis |  |
| User of corticosteroids or other immunosuppressive medications |  |
| Alpha 1 antitrypsin deficiency |  |
| Bronchopulmonary dysplasia |  |
| Hepatitis B |  |
| Hepatitis C |  |
| Thalassemia and Sickle Cell Disease |  |
| Current chemotherapy use |  |
| Auto-immune disorders |  |
| Multiple Sclerosis |  |
| Inflammatory bowel disease |  |
| Ischemic Heart Disease |  |

**Additional Results**

**Table S1:** Phase 1 vs Phase 2 Performance

|  | **All ages** | | **Age 18–64-year-olds** | |
| --- | --- | --- | --- | --- |
|  | **Phase 1** | **Phase 2** | **Phase 1** | **Phase 2** |
| **AUC** | 0.830 (0.821, 0.842) | 0.825 (0.813, 0.836) | 0.802 (0.787, 0.816) | 0.802 (0.787, 0.818) |
| **TPR** |  |  |  |  |
| >=90th | 0.500 (0.476, 0.527) | 0.484 (0.459, 0.511) | 0.448 (0.418, 0.478) | 0.470 (0.434, 0.501) |
| >=75th | 0.761 (0.741, 0.786) | 0.756 (0.732, 0.778) | 0.729 (0.699, 0.755) | 0.724 (0.693, 0.754) |
| >=50th | 0.905 (0.892, 0.922) | 0.900 (0.884, 0.914) | 0.881 (0.858, 0.900) | 0.885 (0.863, 0.906) |
| **FPR** |  |  |  |  |
| >=90th | 0.091 (0.089, 0.093) | 0.092 (0.090, 0.094) | 0.094 (0.092, 0.096) | 0.094 (0.092, 0.097) |
| >=75th | 0.239 (0.236, 0.242) | 0.239 (0.236, 0.243) | 0.242 (0.238, 0.245) | 0.243 (0.239, 0.246) |
| >=50th | 0.491 (0.488, 0.495) | 0.491 (0.488, 0.495) | 0.502 (0.498, 0.507) | 0.494 (0.490, 0.498) |
| **PPV** |  |  |  |  |
| >=90th | 0.107 (0.101, 0.114) | 0.099 (0.092, 0.106) | 0.069 (0.064, 0.073) | 0.071 (0.065, 0.076) |
| >=75th | 0.065 (0.062, 0.069) | 0.062 (0.058, 0.065) | 0.044 (0.043, 0.046) | 0.044 (0.042, 0.045) |
| >=50th | 0.039 (0.037, 0.041) | 0.037 (0.035, 0.039) | 0.026 (0.026, 0.027) | 0.027 (0.026, 0.027) |

Note: Phase 1 models were estimated with all potential predictors defined in KPWHRI’s virtual data warehouse using logistic regression with LASSO for variable selection. Phase 2 models used the predictors selected in Phase 1 only and were estimated using ridge regression

**Table S2a:** Characteristics of infections among 18-64-year-olds in racial and ethnic subgroups – American Indian and Alaskan Native, Asian, Black

|  | **American Indian and Alaskan Native** | | | | **Asian** | | | | **Black** | | | |
| --- | --- | --- | --- | --- | --- | --- | --- | --- | --- | --- | --- | --- |
|  | All | No event | Event | Event rate^1^ | All | No event | Event | Event rate^1^ | All | No event | Event | Event rate^1^ |
|  | N=863 | N=839 | N=24 |  | N=6,463 | N=6,371 | N=92 |  | N=3,952 | N=3,885 | N=67 |  |
| **Age (years)** |  |  |  |  |  |  |  |  |  |  |  |  |
| 18-29 | 188 (21.8%) | 185 (22.1%) | 3 (12.5%) | 16 | 1347 (20.8%) | 1337 (21.0%) | 10 (10.9%) | 7.4 | 875 (22.1%) | 869 (22.4%) | 6 (9.0%) | 6.9 |
| 30-39 | 196 (22.7%) | 191 (22.8%) | 5 (20.8%) | 25.5 | 1733 (26.8%) | 1705 (26.8%) | 28 (30.4%) | 16.2 | 1002 (25.4%) | 983 (25.3%) | 19 (28.4%) | 19 |
| 40-49 | 196 (22.7%) | 188 (22.4%) | 8 (33.3%) | 40.8 | 1523 (23.6%) | 1501 (23.6%) | 22 (23.9%) | 14.4 | 887 (22.4%) | 868 (22.3%) | 19 (28.4%) | 21.4 |
| 50-59 | 202 (23.4%) | 196 (23.4%) | 6 (25.0%) | 29.7 | 1299 (20.1%) | 1275 (20.0%) | 24 (26.1%) | 18.5 | 849 (21.5%) | 833 (21.4%) | 16 (23.9%) | 18.8 |
| 60-69 | 81 (9.4%) | 79 (9.4%) | 2 (8.3%) | 24.7 | 561 (8.7%) | 553 (8.7%) | 8 (8.7%) | 14.3 | 339 (8.6%) | 332 (8.5%) | 7 (10.4%) | 20.6 |
| **Female** | 533 (61.8%) | 523 (62.3%) | 10 (41.7%) | 18.8 | 4082 (63.2%) | 4027 (63.2%) | 55 (59.8%) | 13.5 | 2388 (60.4%) | 2356 (60.6%) | 32 (47.8%) | 13.4 |
| **Male** | 330 (38.2%) | 316 (37.7%) | 14 (58.3%) | 42.4 | 2381 (36.8%) | 2344 (36.8%) | 37 (40.2%) | 15.5 | 1564 (39.6%) | 1529 (39.4%) | 35 (52.2%) | 22.4 |
| **Race**^2^ |  |  |  |  |  |  |  |  |  |  |  |  |
| American Indian/ Alaskan Native | 863 (100.0%) | 839 (100.0%) | 24 (100.0%) | 27.8 | 91 (1.4%) | 88 (1.4%) | 3 (3.3%) | 33 | 91 (2.3%) | 88 (2.3%) | 3 (4.5%) | 33 |
| Asian | 91 (10.5%) | 88 (10.5%) | 3 (12.5%) | 33 | 6463 (100.0%) | 6371 (100.0%) | 92 (100.0%) | 14.2 | 199 (5.0%) | 194 (5.0%) | 5 (7.5%) | 25.1 |
| Black | 91 (10.5%) | 88 (10.5%) | 3 (12.5%) | 33 | 199 (3.1%) | 194 (3.0%) | 5 (5.4%) | 25.1 | 3952 (100.0%) | 3885 (100.0%) | 67 (100.0%) | 17 |
| Native Hawaiian/ Pacific Islander | 24 (2.8%) | 24 (2.9%) | 0 (0.0%) | 0 | 27 (0.4%) | 26 (0.4%) | 1 (1.1%) | 37 | 27 (0.7%) | 26 (0.7%) | 1 (1.5%) | 37 |
| White non-Hispanic | 0 (0.0%) | 0 (0.0%) | 0 (0.0%) |  | 0 (0.0%) | 0 (0.0%) | 0 (0.0%) |  | 0 (0.0%) | 0 (0.0%) | 0 (0.0%) |  |
| **Hispanic ethnicity** | 156 (18.1%) | 152 (18.1%) | 4 (16.7%) | 25.6 | 150 (2.3%) | 148 (2.3%) | 2 (2.2%) | 13.3 | 149 (3.8%) | 147 (3.8%) | 2 (3.0%) | 13.4 |
| **No recorded race or ethnicity** | 0 (0.0%) | 0 (0.0%) | 0 (0.0%) |  | 0 (0.0%) | 0 (0.0%) | 0 (0.0%) |  | 0 (0.0%) | 0 (0.0%) | 0 (0.0%) |  |
| **Medicare insurance** | 26 (3.0%) | 24 (2.9%) | 2 (8.3%) | 76.9 | 53 (0.8%) | 50 (0.8%) | 3 (3.3%) | 56.6 | 76 (1.9%) | 73 (1.9%) | 3 (4.5%) | 39.5 |
| **Length of prior KPWA insurance enrollment** |  |  |  |  |  |  |  |  |  |  |  |  |
| <1 year | 187 (21.7%) | 182 (21.7%) | 5 (20.8%) | 26.7 | 1319 (20.4%) | 1298 (20.4%) | 21 (22.8%) | 15.9 | 923 (23.4%) | 908 (23.4%) | 15 (22.4%) | 16.3 |
| 1-3 years | 255 (29.5%) | 247 (29.4%) | 8 (33.3%) | 31.4 | 1860 (28.8%) | 1838 (28.8%) | 22 (23.9%) | 11.8 | 1155 (29.2%) | 1131 (29.1%) | 24 (35.8%) | 20.8 |
| 3 years or more | 421 (48.8%) | 410 (48.9%) | 11 (45.8%) | 26.1 | 3284 (50.8%) | 3235 (50.8%) | 49 (53.3%) | 14.9 | 1874 (47.4%) | 1846 (47.5%) | 28 (41.8%) | 14.9 |
| **Calendar year** |  |  |  |  |  |  |  |  |  |  |  |  |
| 2020 | 132 (15.3%) | 124 (14.8%) | 8 (33.3%) | 60.6 | 806 (12.5%) | 771 (12.1%) | 35 (38.0%) | 43.4 | 776 (19.6%) | 753 (19.4%) | 23 (34.3%) | 29.6 |
| 2021 | 218 (25.3%) | 206 (24.6%) | 12 (50.0%) | 55 | 1302 (20.1%) | 1260 (19.8%) | 42 (45.7%) | 32.3 | 1078 (27.3%) | 1043 (26.8%) | 35 (52.2%) | 32.5 |
| 2022 | 513 (59.4%) | 509 (60.7%) | 4 (16.7%) | 7.8 | 4355 (67.4%) | 4340 (68.1%) | 15 (16.3%) | 3.4 | 2098 (53.1%) | 2089 (53.8%) | 9 (13.4%) | 4.3 |
| **First documentation of infection**^3^ |  |  |  |  |  |  |  |  |  |  |  |  |
| COVID-19 diagnosis | 227 (26.3%) | 218 (26.0%) | 9 (37.5%) | 39.6 | 1776 (27.5%) | 1744 (27.4%) | 32 (34.8%) | 18 | 1071 (27.1%) | 1049 (27.0%) | 22 (32.8%) | 20.5 |
| Positive PCR test | 586 (67.9%) | 565 (67.3%) | 21 (87.5%) | 35.8 | 4164 (64.4%) | 4098 (64.3%) | 66 (71.7%) | 15.9 | 2875 (72.7%) | 2822 (72.6%) | 53 (79.1%) | 18.4 |
| Positive at-home test | 103 (11.9%) | 103 (12.3%) | 0 (0.0%) | 0 | 892 (13.8%) | 892 (14.0%) | 0 (0.0%) | 0 | 282 (7.1%) | 282 (7.3%) | 0 (0.0%) | 0 |
| **Number of COVID-19 vaccines** |  |  |  |  |  |  |  |  |  |  |  |  |
| 0 | 305 (35.3%) | 286 (34.1%) | 19 (79.2%) | 62.3 | 1428 (22.1%) | 1370 (21.5%) | 58 (63.0%) | 40.6 | 1485 (37.6%) | 1434 (36.9%) | 51 (76.1%) | 34.3 |
| 1 | 43 (5.0%) | 43 (5.1%) | 0 (0.0%) | 0 | 257 (4.0%) | 250 (3.9%) | 7 (7.6%) | 27.2 | 223 (5.6%) | 218 (5.6%) | 5 (7.5%) | 22.4 |
| 2 | 266 (30.8%) | 263 (31.3%) | 3 (12.5%) | 11.3 | 1844 (28.5%) | 1827 (28.7%) | 17 (18.5%) | 9.2 | 1350 (34.2%) | 1340 (34.5%) | 10 (14.9%) | 7.4 |
| 3 or more | 249 (28.9%) | 247 (29.4%) | 2 (8.3%) | 8 | 2934 (45.4%) | 2924 (45.9%) | 10 (10.9%) | 3.4 | 894 (22.6%) | 893 (23.0%) | 1 (1.5%) | 1.1 |

^1^ Rate per 1,000 infections of hospitalization or death within subset of sample with that characteristic (e.g., infections in female members) reported for categorical variables

^2^ Race and ethnicity data are collected by member self-report and follow United States Office of Management of Budget designations.

^3^ Categories are not mutually exclusive as infections could be documented by more than one route on the index date.

**Table S2b**: Characteristics of infections among 18-64-year-olds in racial and ethnic subgroups – Hispanic, Native Hawaiian and Pacific Islander, White non-Hispanic

|  | **Hispanic** | | | | **Native Hawaiian/ Pacific Islander** | | | | **White non-Hispanic** | | | |
| --- | --- | --- | --- | --- | --- | --- | --- | --- | --- | --- | --- | --- |
|  | All | No event | Event | Event rate^1^ | All | No event | Event | Event rate^1^ | All | No event | Event | Event rate^1^ |
|  | N=4,785 | N=4,702 | N=83 |  | N=1,224 | N=1,197 | N=27 |  | N=31,596 | N=31,159 | N=437 |  |
| **Age (years)** |  |  |  |  |  |  |  |  |  |  |  |  |
| 18-29 | 1183 (24.7%) | 1169 (24.9%) | 14 (16.9%) | 11.8 | 271 (22.1%) | 269 (22.5%) | 2 (7.4%) | 7.4 | 5264 (16.7%) | 5235 (16.8%) | 29 (6.6%) | 5.5 |
| 30-39 | 1334 (27.9%) | 1315 (28.0%) | 19 (22.9%) | 14.2 | 320 (26.1%) | 316 (26.4%) | 4 (14.8%) | 12.5 | 7184 (22.7%) | 7123 (22.9%) | 61 (14.0%) | 8.5 |
| 40-49 | 1057 (22.1%) | 1035 (22.0%) | 22 (26.5%) | 20.8 | 341 (27.9%) | 334 (27.9%) | 7 (25.9%) | 20.5 | 7162 (22.7%) | 7077 (22.7%) | 85 (19.5%) | 11.9 |
| 50-59 | 859 (18.0%) | 842 (17.9%) | 17 (20.5%) | 19.8 | 209 (17.1%) | 200 (16.7%) | 9 (33.3%) | 43.1 | 7797 (24.7%) | 7648 (24.5%) | 149 (34.1%) | 19.1 |
| 60-69 | 352 (7.4%) | 341 (7.3%) | 11 (13.3%) | 31.2 | 83 (6.8%) | 78 (6.5%) | 5 (18.5%) | 60.2 | 4189 (13.3%) | 4076 (13.1%) | 113 (25.9%) | 27 |
| **Female** | 2941 (61.5%) | 2889 (61.4%) | 52 (62.7%) | 17.7 | 734 (60.0%) | 720 (60.2%) | 14 (51.9%) | 19.1 | 18454 (58.4%) | 18221 (58.5%) | 233 (53.3%) | 12.6 |
| **Male** | 1844 (38.5%) | 1813 (38.6%) | 31 (37.3%) | 16.8 | 490 (40.0%) | 477 (39.8%) | 13 (48.1%) | 26.5 | 13142 (41.6%) | 12938 (41.5%) | 204 (46.7%) | 15.5 |
| **Race**^2^ |  |  |  |  |  |  |  |  |  |  |  |  |
| American Indian/ Alaskan Native | 156 (3.3%) | 152 (3.2%) | 4 (4.8%) | 25.6 | 24 (2.0%) | 24 (2.0%) | 0 (0.0%) | 0 | 0 (0.0%) | 0 (0.0%) | 0 (0.0%) |  |
| Asian | 150 (3.1%) | 148 (3.1%) | 2 (2.4%) | 13.3 | 27 (2.2%) | 26 (2.2%) | 1 (3.7%) | 37 | 0 (0.0%) | 0 (0.0%) | 0 (0.0%) |  |
| Black | 149 (3.1%) | 147 (3.1%) | 2 (2.4%) | 13.4 | 27 (2.2%) | 26 (2.2%) | 1 (3.7%) | 37 | 0 (0.0%) | 0 (0.0%) | 0 (0.0%) |  |
| Native Hawaiian/ Pacific Islander | 70 (1.5%) | 70 (1.5%) | 0 (0.0%) | 0 | 1224 (100.0%) | 1197 (100.0%) | 27 (100.0%) | 22.1 | 0 (0.0%) | 0 (0.0%) | 0 (0.0%) |  |
| White non-Hispanic | 0 (0.0%) | 0 (0.0%) | 0 (0.0%) |  | 0 (0.0%) | 0 (0.0%) | 0 (0.0%) |  | 31596 (100.0%) | 31159 (100.0%) | 437 (100.0%) | 13.8 |
| **Hispanic ethnicity** | 4785 (100.0%) | 4702 (100.0%) | 83 (100.0%) | 17.3 | 70 (5.7%) | 70 (5.8%) | 0 (0.0%) | 0 | 0 (0.0%) | 0 (0.0%) | 0 (0.0%) |  |
| **No recorded race or ethnicity** | 0 (0.0%) | 0 (0.0%) | 0 (0.0%) |  | 0 (0.0%) | 0 (0.0%) | 0 (0.0%) |  | 0 (0.0%) | 0 (0.0%) | 0 (0.0%) |  |
| **Medicare insurance** | 46 (1.0%) | 43 (0.9%) | 3 (3.6%) | 65.2 | 23 (1.9%) | 21 (1.8%) | 2 (7.4%) | 87 | 673 (2.1%) | 633 (2.0%) | 40 (9.2%) | 59.4 |
| **Length of prior KPWA insurance enrollment** |  |  |  |  |  |  |  |  |  |  |  |  |
| <1 year | 1187 (24.8%) | 1163 (24.7%) | 24 (28.9%) | 20.2 | 228 (18.6%) | 224 (18.7%) | 4 (14.8%) | 17.5 | 6833 (21.6%) | 6719 (21.6%) | 114 (26.1%) | 16.7 |
| 1-3 years | 1601 (33.5%) | 1574 (33.5%) | 27 (32.5%) | 16.9 | 373 (30.5%) | 366 (30.6%) | 7 (25.9%) | 18.8 | 9107 (28.8%) | 8992 (28.9%) | 115 (26.3%) | 12.6 |
| 3 years or more | 1997 (41.7%) | 1965 (41.8%) | 32 (38.6%) | 16 | 623 (50.9%) | 607 (50.7%) | 16 (59.3%) | 25.7 | 15656 (49.6%) | 15448 (49.6%) | 208 (47.6%) | 13.3 |
| **Calendar year** |  |  |  |  |  |  |  |  |  |  |  |  |
| 2020 | 866 (18.1%) | 837 (17.8%) | 29 (34.9%) | 33.5 | 224 (18.3%) | 220 (18.4%) | 4 (14.8%) | 17.9 | 4286 (13.6%) | 4187 (13.4%) | 99 (22.7%) | 23.1 |
| 2021 | 1160 (24.2%) | 1125 (23.9%) | 35 (42.2%) | 30.2 | 383 (31.3%) | 366 (30.6%) | 17 (63.0%) | 44.4 | 8328 (26.4%) | 8090 (26.0%) | 238 (54.5%) | 28.6 |
| 2022 | 2759 (57.7%) | 2740 (58.3%) | 19 (22.9%) | 6.9 | 617 (50.4%) | 611 (51.0%) | 6 (22.2%) | 9.7 | 18982 (60.1%) | 18882 (60.6%) | 100 (22.9%) | 5.3 |
| **First documentation of infection**^3^ |  |  |  |  |  |  |  |  |  |  |  |  |
| COVID-19 diagnosis | 1246 (26.0%) | 1208 (25.7%) | 38 (45.8%) | 30.5 | 323 (26.4%) | 311 (26.0%) | 12 (44.4%) | 37.2 | 8444 (26.7%) | 8312 (26.7%) | 132 (30.2%) | 15.6 |
| Positive PCR test | 3360 (70.2%) | 3297 (70.1%) | 63 (75.9%) | 18.7 | 881 (72.0%) | 864 (72.2%) | 17 (63.0%) | 19.3 | 19622 (62.1%) | 19293 (61.9%) | 329 (75.3%) | 16.8 |
| Positive at-home test | 496 (10.4%) | 495 (10.5%) | 1 (1.2%) | 2 | 94 (7.7%) | 93 (7.8%) | 1 (3.7%) | 10.6 | 5327 (16.9%) | 5322 (17.1%) | 5 (1.1%) | 0.9 |
| **Number of COVID-19 vaccines** |  |  |  |  |  |  |  |  |  |  |  |  |
| 0 | 1638 (34.2%) | 1579 (33.6%) | 59 (71.1%) | 36 | 519 (42.4%) | 498 (41.6%) | 21 (77.8%) | 40.5 | 9964 (31.5%) | 9671 (31.0%) | 293 (67.0%) | 29.4 |
| 1 | 285 (6.0%) | 281 (6.0%) | 4 (4.8%) | 14 | 73 (6.0%) | 72 (6.0%) | 1 (3.7%) | 13.7 | 1819 (5.8%) | 1787 (5.7%) | 32 (7.3%) | 17.6 |
| 2 | 1483 (31.0%) | 1473 (31.3%) | 10 (12.0%) | 6.7 | 362 (29.6%) | 360 (30.1%) | 2 (7.4%) | 5.5 | 8299 (26.3%) | 8236 (26.4%) | 63 (14.4%) | 7.6 |
| 3 or more | 1379 (28.8%) | 1369 (29.1%) | 10 (12.0%) | 7.3 | 270 (22.1%) | 267 (22.3%) | 3 (11.1%) | 11.1 | 11514 (36.4%) | 11465 (36.8%) | 49 (11.2%) | 4.3 |

^1^ Rate per 1,000 infections of hospitalization or death within subset of sample with that characteristic (e.g., infections in female members) reported for categorical variables

^2^ Race and ethnicity data are collected by member self-report and follow United States Office of Management of Budget designations.

^3^ Categories are not mutually exclusive as infections could be documented by more than one route on the index date.

**Table 3a:** Model predictors for infections among 18-64-year-olds in racial and ethnic subgroups – American Indian and Alaskan Native, Asian, Black

|  | **American Indian/Alaskan Native** | | | | **Asian** | | | | **Black** | | | |
| --- | --- | --- | --- | --- | --- | --- | --- | --- | --- | --- | --- | --- |
|  | **All** | **No event** | **Event** | **Event rate**^1^ | **All** | **No event** | **Event** | **Event rate**^1^ | **All** | **No event** | **Event** | **Event rate**^1^ |
|  | N=863 | N=839 | N=24 |  | N=6,463 | N=6,371 | N=92 |  | N=3,952 | N=3,885 | N=67 |  |
| Age, years^2^ | 41.7 (12.9) | 41.6 (12.9) | 46.2 (11.2) |  | 41.1 (12.5) | 41.1 (12.5) | 44.2 (11.8) |  | 41 (12.7) | 40.9 (12.7) | 44.3 (11.6) |  |
| Documented race or ethnicity other than White non-Hispanic^3^ | 863 (100.0%) | 839 (100.0%) | 24 (100.0%) | 27.8 | 6463 (100.0%) | 6371 (100.0%) | 92 (100.0%) | 14.2 | 3952 (100.0%) | 3885 (100.0%) | 67 (100.0%) | 17 |
| ACG 12-month hospitalization risk | 0.38 (0.59) | 0.37 (0.56) | 0.74 (1.09) |  | 0.2 (0.28) | 0.2 (0.27) | 0.42 (0.66) |  | 0.3 (0.51) | 0.3 (0.49) | 0.63 (1.2) |  |
| ACG missing ^4^ | 42 (4.9%) | 42 (5.0%) | 0 (0.0%) | 0 | 349 (5.4%) | 344 (5.4%) | 5 (5.4%) | 14.3 | 228 (5.8%) | 226 (5.8%) | 2 (3.0%) | 8.8 |
| >10 outpatient visits in prior year | 61 (7.1%) | 58 (6.9%) | 3 (12.5%) | 49.2 | 339 (5.2%) | 326 (5.1%) | 13 (14.1%) | 38.3 | 259 (6.6%) | 254 (6.5%) | 5 (7.5%) | 19.3 |
| BMI ≥ 30 kg/m^2^ | 496 (57.5%) | 480 (57.2%) | 16 (66.7%) | 32.3 | 1649 (25.5%) | 1606 (25.2%) | 43 (46.7%) | 26.1 | 2134 (54.0%) | 2090 (53.8%) | 44 (65.7%) | 20.6 |
| Diagnoses |  |  |  |  |  |  |  |  |  |  |  |  |
| Autoimmune diagnosis | 23 (2.7%) | 23 (2.7%) | 0 (0.0%) | 0 | 140 (2.2%) | 140 (2.2%) | 0 (0.0%) | 0 | 91 (2.3%) | 91 (2.3%) | 0 (0.0%) | 0 |
| Chronic kidney disease | 20 (2.3%) | 18 (2.1%) | 2 (8.3%) | 100 | 60 (0.9%) | 54 (0.8%) | 6 (6.5%) | 100 | 77 (1.9%) | 73 (1.9%) | 4 (6.0%) | 51.9 |
| Chronic lung disease | 10 (1.2%) | 10 (1.2%) | 0 (0.0%) | 0 | 12 (0.2%) | 11 (0.2%) | 1 (1.1%) | 83.3 | 33 (0.8%) | 32 (0.8%) | 1 (1.5%) | 30.3 |
| Congestive heart failure | 6 (0.7%) | 6 (0.7%) | 0 (0.0%) | 0 | 18 (0.3%) | 17 (0.3%) | 1 (1.1%) | 55.6 | 31 (0.8%) | 28 (0.7%) | 3 (4.5%) | 96.8 |
| Dementia | 0 (0.0%) | 0 (0.0%) | 0 (0.0%) |  | 0 (0.0%) | 0 (0.0%) | 0 (0.0%) |  | 0 (0.0%) | 0 (0.0%) | 0 (0.0%) |  |
| Diabetes | 117 (13.6%) | 108 (12.9%) | 9 (37.5%) | 76.9 | 600 (9.3%) | 575 (9.0%) | 25 (27.2%) | 41.7 | 450 (11.4%) | 433 (11.1%) | 17 (25.4%) | 37.8 |
| Hepatitis | 5 (0.6%) | 5 (0.6%) | 0 (0.0%) | 0 | 131 (2.0%) | 124 (1.9%) | 7 (7.6%) | 53.4 | 37 (0.9%) | 36 (0.9%) | 1 (1.5%) | 27 |
| Immunosuppressed | 12 (1.4%) | 12 (1.4%) | 0 (0.0%) | 0 | 77 (1.2%) | 76 (1.2%) | 1 (1.1%) | 13 | 62 (1.6%) | 60 (1.5%) | 2 (3.0%) | 32.3 |
| Peripheral vascular disease | 1 (0.1%) | 1 (0.1%) | 0 (0.0%) | 0 | 13 (0.2%) | 10 (0.2%) | 3 (3.3%) | 230.8 | 14 (0.4%) | 14 (0.4%) | 0 (0.0%) | 0 |
| COVID-19 related |  |  |  |  |  |  |  |  |  |  |  |  |
| Infection documented with diagnosis (vs. test) | 227 (26.3%) | 218 (26.0%) | 9 (37.5%) | 39.6 | 1776 (27.5%) | 1744 (27.4%) | 32 (34.8%) | 18 | 1071 (27.1%) | 1049 (27.0%) | 22 (32.8%) | 20.5 |
| No prior vaccination | 305 (35.3%) | 286 (34.1%) | 19 (79.2%) | 62.3 | 1428 (22.1%) | 1370 (21.5%) | 58 (63.0%) | 40.6 | 1485 (37.6%) | 1434 (36.9%) | 51 (76.1%) | 34.3 |
| Prior vaccinations, count | 1.6 (1.3) | 1.6 (1.3) | 0.5 (1) |  | 2 (1.2) | 2 (1.2) | 0.8 (1.1) |  | 1.4 (1.2) | 1.5 (1.2) | 0.4 (0.8) |  |
| Only vaccination within past 2 weeks | 4 (0.5%) | 4 (0.5%) | 0 (0.0%) | 0 | 48 (0.7%) | 43 (0.7%) | 5 (5.4%) | 104.2 | 24 (0.6%) | 23 (0.6%) | 1 (1.5%) | 41.7 |

^1^ Rate per 1,000 infections of hospitalization or death within subset of sample with that characteristic

^2^ For continuous or count variables, the mean(standard deviation) is reported instead of counts and percentages. No event rate is presented.

^3^ Composite binary variable designating members of color. Variable equals 1 for infections in member with some self-reported race or ethnicity other than White non-Hispanic. If no race or ethnicity is reported, this variable equals 0.

^4^ For infections missing ACG, mean imputation was used for prediction model estimation, validation, and implementation

**Table 3b:** Model predictors for infections among 18-64-year-olds in racial and ethnic subgroups Hispanic, Native Hawaiian and Pacific Islander, White non-Hispanic

|  | **Hispanic** | | | | **Native Hawaiian/ Pacific Islander** | | | | **White non-Hispanic** | | | |
| --- | --- | --- | --- | --- | --- | --- | --- | --- | --- | --- | --- | --- |
|  | **All** | **No event** | **Event** | **Event rate**^1^ | **All** | **No event** | **Event** | **Event rate**^1^ | **All** | **No event** | **Event** | **Event rate**^1^ |
|  | N=4,785 | N=4,702 | N=83 |  | N=1,224 | N=1,197 | N=27 |  | N=31,596 | N=31,159 | N=437 |  |
| Age, years^2^ | 39.7 (12.3) | 39.6 (12.3) | 43.4 (12.1) |  | 40 (12) | 39.8 (12) | 47.6 (11.1) |  | 43.7 (12.7) | 43.6 (12.7) | 49.9 (11.4) |  |
| Documented race or ethnicity other than White non-Hispanic^3^ | 4785 (100.0%) | 4702 (100.0%) | 83 (100.0%) | 17.3 | 1224 (100.0%) | 1197 (100.0%) | 27 (100.0%) | 22.1 | 0 (0.0%) | 0 (0.0%) | 0 (0.0%) |  |
| ACG 12-month hospitalization risk | 0.26 (0.4) | 0.25 (0.38) | 0.57 (0.84) |  | 0.29 (0.48) | 0.28 (0.46) | 0.63 (1) |  | 0.3 (0.46) | 0.3 (0.43) | 0.76 (1.18) |  |
| ACG missing ^4^ | 326 (6.8%) | 320 (6.8%) | 6 (7.2%) | 18.4 | 47 (3.8%) | 46 (3.8%) | 1 (3.7%) | 21.3 | 1377 (4.4%) | 1361 (4.4%) | 16 (3.7%) | 11.6 |
| >10 outpatient visits in prior year | 306 (6.4%) | 290 (6.2%) | 16 (19.3%) | 52.3 | 56 (4.6%) | 53 (4.4%) | 3 (11.1%) | 53.6 | 1883 (6.0%) | 1827 (5.9%) | 56 (12.8%) | 29.7 |
| BMI ≥ 30 kg/m^2^ | 2377 (49.7%) | 2321 (49.4%) | 56 (67.5%) | 23.6 | 738 (60.3%) | 718 (60.0%) | 20 (74.1%) | 27.1 | 14828 (46.9%) | 14537 (46.7%) | 291 (66.6%) | 19.6 |
| **Diagnoses** |  |  |  |  |  |  |  |  |  |  |  |  |
| Autoimmune diagnosis | 112 (2.3%) | 109 (2.3%) | 3 (3.6%) | 26.8 | 28 (2.3%) | 27 (2.3%) | 1 (3.7%) | 35.7 | 803 (2.5%) | 782 (2.5%) | 21 (4.8%) | 26.2 |
| Chronic kidney disease | 50 (1.0%) | 48 (1.0%) | 2 (2.4%) | 40 | 41 (3.3%) | 36 (3.0%) | 5 (18.5%) | 122 | 346 (1.1%) | 316 (1.0%) | 30 (6.9%) | 86.7 |
| Chronic lung disease | 16 (0.3%) | 15 (0.3%) | 1 (1.2%) | 62.5 | 9 (0.7%) | 8 (0.7%) | 1 (3.7%) | 111.1 | 304 (1.0%) | 293 (0.9%) | 11 (2.5%) | 36.2 |
| Congestive heart failure | 15 (0.3%) | 14 (0.3%) | 1 (1.2%) | 66.7 | 20 (1.6%) | 17 (1.4%) | 3 (11.1%) | 150 | 186 (0.6%) | 169 (0.5%) | 17 (3.9%) | 91.4 |
| Dementia | 3 (0.1%) | 2 (0.0%) | 1 (1.2%) | 333.3 | 0 (0.0%) | 0 (0.0%) | 0 (0.0%) |  | 2 (0.0%) | 2 (0.0%) | 0 (0.0%) | 0 |
| Diabetes | 469 (9.8%) | 449 (9.5%) | 20 (24.1%) | 42.6 | 193 (15.8%) | 186 (15.5%) | 7 (25.9%) | 36.3 | 2391 (7.6%) | 2300 (7.4%) | 91 (20.8%) | 38.1 |
| Hepatitis | 34 (0.7%) | 33 (0.7%) | 1 (1.2%) | 29.4 | 10 (0.8%) | 9 (0.8%) | 1 (3.7%) | 100 | 171 (0.5%) | 165 (0.5%) | 6 (1.4%) | 35.1 |
| Immunosuppressed | 64 (1.3%) | 59 (1.3%) | 5 (6.0%) | 78.1 | 26 (2.1%) | 23 (1.9%) | 3 (11.1%) | 115.4 | 589 (1.9%) | 574 (1.8%) | 15 (3.4%) | 25.5 |
| Peripheral vascular disease | 5 (0.1%) | 5 (0.1%) | 0 (0.0%) | 0 | 3 (0.2%) | 2 (0.2%) | 1 (3.7%) | 333.3 | 111 (0.4%) | 102 (0.3%) | 9 (2.1%) | 81.1 |
| **COVID-19 related** |  |  |  |  |  |  |  |  |  |  |  |  |
| Infection documented with diagnosis (vs. test) | 1246 (26.0%) | 1208 (25.7%) | 38 (45.8%) | 30.5 | 323 (26.4%) | 311 (26.0%) | 12 (44.4%) | 37.2 | 8444 (26.7%) | 8312 (26.7%) | 132 (30.2%) | 15.6 |
| No prior vaccination | 1638 (34.2%) | 1579 (33.6%) | 59 (71.1%) | 36 | 519 (42.4%) | 498 (41.6%) | 21 (77.8%) | 40.5 | 9964 (31.5%) | 9671 (31.0%) | 293 (67.0%) | 29.4 |
| Prior vaccinations, count | 1.6 (1.3) | 1.6 (1.3) | 0.7 (1.2) |  | 1.3 (1.3) | 1.4 (1.3) | 0.5 (1.1) |  | 1.7 (1.4) | 1.8 (1.3) | 0.7 (1.1) |  |
| Only vaccination within past 2 weeks | 37 (0.8%) | 36 (0.8%) | 1 (1.2%) | 27 | 20 (1.6%) | 19 (1.6%) | 1 (3.7%) | 50 | 267 (0.8%) | 253 (0.8%) | 14 (3.2%) | 52.4 |

^1^ Rate per 1,000 infections of hospitalization or death within subset of sample with that characteristic

^2^ For continuous or count variables, the mean(standard deviation) is reported instead of counts and percentages. No event rate is presented.

^3^ Composite binary variable designating members of color. Variable equals 1 for infections in member with some self-reported race or ethnicity other than White non-Hispanic. If no race or ethnicity is reported, this variable equals 0.

^4^ For infections missing ACG, mean imputation was used for prediction model estimation, validation, and implementation

Table S4: Prediction model performance across racial and ethnic subgroups for infections in 18-64-year-olds

|  | **American Indian/Alaskan Native** | **Asian** | **Black/ African American** | **Hispanic** | **Native Hawaiian/ Pacific Islander** | **White non-Hispanic** | **Not recorded** |
| --- | --- | --- | --- | --- | --- | --- | --- |
| **AUC** | 0.831 (0.763, 0.898) | 0.811 (0.765, 0.859) | 0.778 (0.720, 0.829) | 0.791 (0.737, 0.841) | 0.827 (0.776, 0.873) | 0.807 (0.788, 0.828) | 0.791 (0.736, 0.841) |
| **TPR** |  |  |  |  |  |  |  |
| ≥90^th^ | 0.667 (0.533, 0.800) | 0.506 (0.414, 0.602) | 0.490 (0.376, 0.613) | 0.508 (0.395, 0.621) | 0.630 (0.493, 0.760) | 0.480 (0.430, 0.527) | 0.290 (0.190 0.393) |
| ≥75^th^ | 0.817 (0.683, 0.950) | 0.660 (0.571, 0.750) | 0.814 (0.706, 0.906) | 0.771 (0.680, 0.856) | 0.813 (0.727, 0.900) | 0.723 (0.681, 0.763) | 0.611 (0.503, 0.714) |
| ≥50^th^ | 0.967 (0.900, 1) | 0.820 (0.737, 0.897) | 0.928 (0.864, 0.978) | 0.872 (0.797, 0.941) | 0.980 (0.940, 1) | 0.890 (0.859, 0.919) | 0.840 (0.756, 0.916) |
| **FPR** |  |  |  |  |  |  |  |
| ≥90^th^ | 0.152 (0.127, 0.176) | 0.066 (0.060, 0.072) | 0.148 (0.137, 0.159) | 0.117 (0.109, 0.127) | 0.177 (0.156, 0.198) | 0.092 (0.089, 0.095) | 0.043 (0.037, 0.049) |
| ≥75^th^ | 0.324 (0.293, 0.355) | 0.183 (0.173, 0.192) | 0.321 (0.308, 0.336) | 0.287 (0.274, 0.300) | 0.388 (0.361, 0.413) | 0.240 (0.235, 0.244) | 0.175 (0.164, 0.187) |
| ≥50^th^ | 0.615 (0.581, 0.649) | 0.391 (0.379, 0.403) | 0.633 (0.617, 0.648) | 0.549 (0.535, 0.564) | 0.661 (0.635, 0.686) | 0.488 (0.483, 0.494) | 0.438 (0.423, 0.453) |
| **PPV** |  |  |  |  |  |  |  |
| ≥90^th^ | 0.104 (0.077, 0.141) | 0.101 (0.083, 0.122) | 0.054 (0.042, 0.067) | 0.071 (0.057, 0.088) | 0.071 (0.051, 0.097) | 0.068 (0.061, 0.075) | 0.101 (0.067, 0.140) |
| ≥75^th^ | 0.069 (0.058, 0.083) | 0.051 (0.045, 0.058) | 0.042 (0.036, 0.047) | 0.045 (0.040, 0.051) | 0.047 (0.041, 0.054) | 0.041 (0.038, 0.043) | 0.055 (0.045, 0.065) |
| ≥50^th^ | 0.042 (0.039, 0.046) | 0.030 (0.027, 0.033) | 0.025 (0.023, 0.026) | 0.027 (0.025, 0.03) | 0.032 (0.029, 0.034) | 0.025 (0.024, 0.026) | 0.031 (0.028, 0.034) |

Note: (NPV is not included in Table S4, as estimates were over 0.98 for all thresholds and all subgroups.)

^1^ Thresholds for classification accuracy measures were defined based on the distribution of risk scores among all infections. For example, ≥ 90^th^ percentile refers to the risk prediction threshold that would classify the 10% of infections with the greatest risk as high-risk for hospitalization and death and, subsequently, classify the 90% of infections with a predicted risk below that threshold not high-risk.

**Table S5:** Characteristics of infections among 18-64-year-olds in threshold assessment analysis

|  | **All** | **No event** | **Event** | **Event rate**^1^ |
| --- | --- | --- | --- | --- |
|  | N=18,437 | N=18,351 | N=86 |  |
| **Age (years)** |  |  |  |  |
| 18-29 | 2659 (14.4%) | 2644 (14.4%) | 15 (17.4%) | 5.6 |
| 30-39 | 4661 (25.3%) | 4635 (25.3%) | 26 (30.2%) | 5.6 |
| 40-49 | 4329 (23.5%) | 4313 (23.5%) | 16 (18.6%) | 3.7 |
| 50-59 | 4344 (23.6%) | 4328 (23.6%) | 16 (18.6%) | 3.7 |
| 60-69 | 2444 (13.3%) | 2431 (13.2%) | 13 (15.1%) | 5.3 |
| **Female** | 11303 (61.3%) | 11247 (61.3%) | 56 (65.1%) | 5 |
| **Male** | 7134 (38.7%) | 7104 (38.7%) | 30 (34.9%) | 4.2 |
| **Race**^2^ |  |  |  |  |
| American Indian/Alaskan Native | 253 (1.4%) | 252 (1.4%) | 1 (1.2%) | 4 |
| Asian | 2636 (14.3%) | 2623 (14.3%) | 13 (15.1%) | 4.9 |
| Black | 1004 (5.4%) | 999 (5.4%) | 5 (5.8%) | 5 |
| Native Hawaiian/Pacific Islander | 275 (1.5%) | 272 (1.5%) | 3 (3.5%) | 10.9 |
| White non-Hispanic | 11293 (61.3%) | 11247 (61.3%) | 46 (53.5%) | 4.1 |
| **Hispanic ethnicity** | 1282 (7.0%) | 1271 (6.9%) | 11 (12.8%) | 8.6 |
| **No recorded race or ethnicity** | 1616 (8.8%) | 1609 (8.8%) | 7 (8.1%) | 4.3 |
| **Medicare insurance** | 306 (1.7%) | 299 (1.6%) | 7 (8.1%) | 22.9 |
| **Length of prior KPWA insurance enrollment** |  |  |  |  |
| <1 year | 4003 (21.7%) | 3976 (21.7%) | 27 (31.4%) | 6.7 |
| 1-3 years | 4989 (27.1%) | 4969 (27.1%) | 20 (23.3%) | 4 |
| 3 years or more | 9445 (51.2%) | 9406 (51.3%) | 39 (45.3%) | 4.1 |
| **Calendar year** |  |  |  |  |
| 2020 | 0 | 0 | 0 | 0 |
| 2021 | 0 | 0 | 0 | 0 |
| 2022 | 18437 (100.0%) | 18351 (100.0%) | 86 (100.0%) | 4.7 |
| **First documentation of infection**^3^ |  |  |  |  |
| COVID-19 diagnosis | 6578 (35.7%) | 6539 (35.6%) | 39 (45.3%) | 5.9 |
| Positive PCR test | 5921 (32.1%) | 5876 (32.0%) | 45 (52.3%) | 7.6 |
| Positive at-home test | 7281 (39.5%) | 7275 (39.6%) | 6 (7.0%) | 0.8 |
| **Number of COVID-19 vaccines** |  |  |  |  |
| 0 | 953 (5.2%) | 945 (5.1%) | 8 (9.3%) | 8.4 |
| 1 | 575 (3.1%) | 572 (3.1%) | 3 (3.5%) | 5.2 |
| 2 | 3738 (20.3%) | 3711 (20.2%) | 27 (31.4%) | 7.2 |
| 3 or more | 13171 (71.4%) | 13123 (71.5%) | 48 (55.8%) | 3.6 |

^1^ Rate per 1,000 infections of hospitalization or death within subset of sample with that characteristic (e.g., infections in female members) reported for categorical variables

^2^ Race and ethnicity data are collected by member self-report and follow United States Office of Management of Budget designations.

^3^ Categories are not mutually exclusive as infections could be documented by more than one route on the index date.

**Table S6:** Distribution of model predictors among 18-64-year-olds in threshold assessment analysis

|  | **All** | **No event** | **Event** | **Event rate**^1^ |
| --- | --- | --- | --- | --- |
|  | N=18,437 | N=18,351 | N=86 |  |
|  |  |  |  |  |
| Age, years^2^ | 43.8 (12.3) | 43.8 (12.3) | 42.6 (12.8) |  |
| Documented race or ethnicity other than White non-Hispanic^3^ | 5528 (30.0%) | 5495 (29.9%) | 33 (38.4%) | 6 |
| ACG 12-month hospitalization risk | 0.27 (0.37) | 0.27 (0.36) | 0.59 (0.96) |  |
| ACG missing^4^ | 702 (3.8%) | 698 (3.8%) | 4 (4.7%) | 5.7 |
| >10 outpatient visits in prior year | 1320 (7.2%) | 1294 (7.1%) | 26 (30.2%) | 19.7 |
| BMI ≥ 30 kg/m^2^ | 7996 (43.4%) | 7948 (43.3%) | 48 (55.8%) | 6 |
| **Diagnoses** |  |  |  |  |
| Autoimmune diagnosis | 451 (2.4%) | 449 (2.4%) | 2 (2.3%) | 4.4 |
| Chronic kidney disease | 213 (1.2%) | 208 (1.1%) | 5 (5.8%) | 23.5 |
| Chronic lung disease | 30 (0.2%) | 30 (0.2%) | 0 (0.0%) | 0 |
| Congestive heart failure | 91 (0.5%) | 88 (0.5%) | 3 (3.5%) | 33 |
| Dementia | 0 (0.0%) | 0 (0.0%) | 0 (0.0%) |  |
| Diabetes | 1601 (8.7%) | 1589 (8.7%) | 12 (14.0%) | 7.5 |
| Hepatitis | 147 (0.8%) | 144 (0.8%) | 3 (3.5%) | 20.4 |
| Immunosuppressed | 253 (1.4%) | 252 (1.4%) | 1 (1.2%) | 4 |
| Peripheral vascular disease | 46 (0.2%) | 44 (0.2%) | 2 (2.3%) | 43.5 |
| **COVID-19 related** |  |  |  |  |
| Infection documented with diagnosis (vs. test) | 6578 (35.7%) | 6539 (35.6%) | 39 (45.3%) | 5.9 |
| No prior vaccination | 953 (5.2%) | 945 (5.1%) | 8 (9.3%) | 8.4 |
| Prior vaccinations, count | 2.7 (0.9) | 2.7 (0.9) | 2.5 (1.1) |  |
| Only vaccination within past 2 weeks | 2 (0.0%) | 2 (0.0%) | 0 (0.0%) | 0 |

^1^ Rate per 1,000 infections of hospitalization or death within subset of sample with that characteristic

^2^ For continuous or count variables, the mean(standard deviation) is reported instead of counts and percentages. No event rate is presented.

^3^ Composite binary variable designating members of color. Variable equals 1 for infections in member with some self-reported race or ethnicity other than White non-Hispanic. If no race or ethnicity is reported, this variable equals 0.

^4^ For infections missing ACG, mean imputation was used for prediction model estimation, validation, and implementation

CHARMS 2014 Checklist

| **Domain** | **Key items** | **Reported on page #** | **Additional details** |
| --- | --- | --- | --- |
| Participants | Source of data | 6-8 | Data collected from KPWA Epic and KPWA Health Research Institute Virtual Data Warehouse |
|  | Participant eligibility and recruitment method | 6 | Recruitment not applicable |
|  | Participant description | Page 12, Table 1 |  |
|  | Details of treatments received, if relevant | n/a |  |
|  | Study dates | 6 |  |
| Outcome(s) to be predicted | Definition and method for measurement of outcome | 7 |  |
|  | Was the same outcome definition (and method for measurement) used in all patients? | 7 | Yes. Not necessary to confirm explicitly in manuscript, as all outcomes were assessed uniformly in EHR and claims data |
|  | Type of outcome (e.g., single or combined endpoints) | 7 |  |
|  | Was the outcome assessed without knowledge of the candidate predictors (i.e., blinded)? | 7 | Yes. Not necessary to confirm explicitly in manuscript, as all outcomes were assessed uniformly in EHR and claims data |
|  | Were candidate predictors part of the outcome (e.g., in panel or consensus diagnosis)? | 7 | No. Not necessary to state explicitly in manuscript as all outcomes were assessed uniformly in EHR and claims data |
|  | Time of outcome occurrence or summary of duration of follow-up | 7 | 14-day follow-up is assumed complete for all members, as all would have documentation of hospitalization or death |
| Candidate Predictors | Number and type of predictors (e.g., demographics, patient history, physical examination,  additional testing, disease characteristics) | Page 7-8 and supplement pages 2-4 |  |
|  | Definition and method for measurement of candidate predictors | Page 7-8 and Supplement pages 2-4 |  |
|  | Timing of predictor measurement (e.g., at patient presentation, at diagnosis, at treatment initiation) | 7-8 |  |
|  | Were predictors assessed blinded for outcome, and for each other (if relevant)? | 7-8 | Yes. Not necessary to confirm explicitly in manuscript as all predictors were assessed uniformly in EHR and claims data |
|  | Handling of predictors in the modelling (e.g., continuous, linear, non-linear transformations or  categorised) | Table 2 |  |
| Sample size | Number of participants and number of outcomes/events | Page 12-13, Table 1 |  |
|  | Number of outcomes/events in relation to the number of candidate predictors (Events Per Variable) | page 7-8, page 12-13, supplement pages 2-4 | 13 EPV |
|  | Number of participants with any missing value (include predictors and outcomes) | Table 2 | 14-day outcome data are not missing for any infection. The only predictor for which missingness is possible is ACG score (and missingness is reported in Table 2) |
|  | Number of participants with missing data for each predictor | Table 2 | The only predictor for which missingness is possible is ACG score (and missingness is reported in Table 2) |
|  | Handling of missing data (e.g., complete-case analysis, imputation, or other methods) | Table 2 | See footnote for ACG score |
| Model development | Modelling method (e.g., logistic, survival, neural network, or machine learning techniques) | 9-10 |  |
|  | Modelling assumptions satisfied | n/a | Unclear which modeling assumptions would be required for logistic regression with binary outcome. As we are not making inference on coefficient estimates, accuracy of model specification (e.g., additive effects vs. interactions for variables) is not needed for valid evaluation of model performance. |
|  | Method for selection of predictors for inclusion in multivariable modelling (e.g., all candidate  predictors, pre-selection based on unadjusted association with the outcome) | 9-10 |  |
|  | Method for selection of predictors during multivariable modelling (e.g., full model approach,  backward or forward selection) and criteria used (e.g., p-value, Akaike Information Criterion) | 9-10 | Penalization factor for LASSO and ridge selected to minimize cross-validated binomial deviance. Performed via cv.glmnet in glmnet R package. |
|  | Shrinkage of predictor weights or regression coefficients (e.g., no shrinkage, uniform shrinkage,  penalized estimation) | 9-10 |  |
| Model performance | Calibration (calibration plot, calibration slope, Hosmer-Lemeshow test) and Discrimination  (C-statistic, D-statistic, log-rank) measures with confidence intervals | Page 13-14, Table 3, Figures 1-3, Table S1, Table S4 |  |
|  | Classification measures (e.g., sensitivity, specificity, predictive values, net reclassification  improvement) and whether a-priori cut points were used | Page 13-14, Table 3, Figures 1-3, Table S1, Table S4 |  |
| Model evaluation | Method used for testing model performance: development dataset only (random split of data,  resampling methods e.g. bootstrap or cross-validation, none) or separate external validation (e.g.  temporal, geographical, different setting, different investigators) | 8 |  |
|  | In case of poor validation, whether model was adjusted or updated (e.g., intercept recalibrated,  predictor effects adjusted, or new predictors added) | n/a |  |
| Results | Final and other multivariable models (e.g., basic, extended, simplified) presented, including  predictor weights or regression coefficients, intercept, baseline survival, model performance  measures (with standard errors or confidence intervals) | Table 2 | Model performance measures cited above under “model performance” |
|  | Any alternative presentation of the final prediction models, e.g., sum score, nomogram, score chart,  predictions for specific risk subgroups with performance | n/a |  |
|  | Comparison of the distribution of predictors (including missing data) for development and  validation datasets | n/a | As data were randomly divided into 10 folds for cross-validation, comparison across 10 folds would be unnecessary and impractical |
| Interpretation and Discussion | Interpretation of presented models (confirmatory, i.e., model useful for practice versus exploratory,  i.e., more research needed) | 13-15 |  |
|  | Comparison with other studies, discussion of generalizability, strengths and limitations. | 15-20 |  |
